# Disruption of a Hedgehog-Foxf1-Rspo2 signaling axis leads to tracheomalacia and a loss of Sox9^+^ tracheal chondrocytes

**DOI:** 10.1242/dmm.046573

**Published:** 2021-02-04

**Authors:** Talia Nasr, Andrea M. Holderbaum, Praneet Chaturvedi, Kunal Agarwal, Jessica L. Kinney, Keziah Daniels, Stephen L. Trisno, Vladimir Ustiyan, John M. Shannon, James M. Wells, Debora Sinner, Vladimir V. Kalinichenko, Aaron M. Zorn

**Affiliations:** 1 Center for Stem Cell and Organoid Medicine, Division of Developmental Biology, Perinatal Institute, Cincinnati Children's Hospital Medical Center, Cincinnati, OH 45229, USA; 2 Department of Pediatrics, University of Cincinnati College of Medicine, Cincinnati, OH 45267, USA; 3 Division of Pulmonary Biology, Cincinnati Children's Hospital Medical Center, Cincinnati, OH 45229; 4 Center for Lung Regenerative Medicine, Perinatal Institute, Cincinnati Children's Hospital Medical Center, Cincinnati, OH 45229, USA

**Keywords:** Trachea, Tracheomalacia, Cartilage, Hedgehog

## Abstract

Congenital tracheomalacia, resulting from incomplete tracheal cartilage development, is a relatively common birth defect that severely impairs breathing in neonates. Mutations in the Hedgehog (HH) pathway and downstream Gli transcription factors are associated with tracheomalacia in patients and mouse models; however, the underlying molecular mechanisms are unclear. Using multiple *HH/Gli* mouse mutants, including one that mimics Pallister–Hall Syndrome, we show that excessive Gli repressor activity prevents specification of tracheal chondrocytes. Lineage-tracing experiments show that Sox9^+^ chondrocytes arise from HH-responsive splanchnic mesoderm in the fetal foregut that expresses the transcription factor Foxf1. Disrupted HH/Gli signaling results in (1) loss of Foxf1, which in turn is required to support Sox9^+^ chondrocyte progenitors, and (2) a dramatic reduction in *Rspo2*, a secreted ligand that potentiates Wnt signaling known to be required for chondrogenesis. These results reveal an HH-Foxf1-Rspo2 signaling axis that governs tracheal cartilage development and informs the etiology of tracheomalacia.

This article has an associated First Person interview with the first author of the paper.

## Abstract (precis)

**Summary:** Genetic and molecular analyses of mutant mouse embryos reveal a HH-Foxf1-Rspo2 signaling axis that informs the mechanistic basis of tracheomalacia in individuals with Hedgehog pathway mutations.

## INTRODUCTION

Impaired formation of the tracheal cartilage, or tracheomalacia, occurs in 1 in 2100 live births and can result in life-threatening airway collapse and impaired breathing (Boogaard et al., 2005; Kamran and Jennings, 2019). Current surgical treatment includes insertion of stents to keep the airway open, but these frequently lead to localized inflammation and multiple subsequent surgeries as the patients age (Fraga et al., 2016; Wallis et al., 2019). Generating biologically accurate replacement tissue from pluripotent stem cells is an aspirational strategy to improve patient care, but this requires a detailed understanding of both normal fetal tracheal development and the etiology of tracheomalacia (Fraga et al., 2016; Wallis et al., 2019).

Tracheal cartilage development in the mouse begins by embryonic day (E)11.5 with expression of the transcription factor Sox9, a master regulator of chondrogenesis, in the ventral and lateral splanchnic mesenchyme surrounding the fetal trachea (Hines et al., 2013). Sox9^+^ cells do not condense around the dorsal side of the trachea, which forms the trachealis smooth muscle. Between E11.5 and E14.5, as the trachea continues to lengthen and grow, the Sox9^+^ presumptive chondrocytes organize into distinct C-shaped rings separated by fibroelastic tissue along the anterior-posterior axis of the trachea (Kishimoto et al., 2018; Park et al., 2010). By E15.5, the chondrocytes differentiate into cartilage rings (Park et al., 2010). Hedgehog (HH) and Wnt signaling are critical for tracheal cartilage development in mice, and mutations in these pathways have been associated with tracheomalacia in patients; however, how these pathways interact to regulate tracheal chondrogenesis is unclear (Sinner et al., 2019).

The transcription factor Sox9 is required for the development of chondrocyte progenitors throughout the body (Lefebvre et al., 2019). Genetic deletion of *Wls*, which encodes the cargo protein essential for Wnt ligand secretion from the tracheal epithelium, leads to a loss of Sox9 expression in the tracheal mesenchyme and a failure in chondrocyte development, causing eventual tracheomalacia (Snowball et al., 2015). Mutations in a number of other Wnt ligands or receptors expressed in the fetal foregut, including *Wnt4*, *Wnt5a*, *Wnt7b*, *Ror2* and *Rspo2*, also display deficits in cartilage development with varying extents of tracheomalacia (Bell et al., 2008; Caprioli et al., 2015; Kishimoto et al., 2018; Li et al., 2002).

Disruption in HH signaling can similarly result in tracheomalacia and loss of Sox9^+^ tracheal chondrocytes in mice (Litingtung et al., 1998; Miller et al., 2004; Motoyama et al., 1998; Park et al., 2010). The HH pathway regulates gene expression via zinc finger Gli transcription factors. In the absence of HH ligands, the HH receptor Smoothened is inhibited, leading to the proteolytical processing of Gli2 and Gli3 into isoforms that act as transcriptional repressors (GliR) (Briscoe and Thérond, 2013). In the presence of HH, Smoothened is active, leading to the production of full-length Gli2 and Gli3 isoforms that activate target gene transcription (GliA). In general, Gli3 predominantly acts in the transcriptional repressor form, whereas Gli2 largely acts as a transcriptional activator (Litingtung et al., 2002; te Welscher et al., 2002; Vokes et al., 2008). Shh ligand is expressed in the developing foregut epithelium in which it signals to the surrounding mesenchyme to regulate Gli activity (Ioannides et al., 2003). In *Shh^−/−^* mutants, the primitive foregut tube fails to separate into distinct trachea and esophagus (Litingtung et al., 1998; Miller et al., 2004; Park et al., 2010). Cartilage never forms around the mutant foregut and there is a dramatic reduction in Sox9 expression and proliferation of the ventral foregut mesenchyme (Litingtung et al., 1998; Miller et al., 2004; Park et al., 2010). *Gli2^−/−^;Gli3^+/−^* mouse embryos that have only one copy of Gli3 also exhibit tracheomalacia, whereas *Gli2^+/−^;Gli3^−/−^* embryos, which lack Gli3 but have a single copy of Gli2, do not (Motoyama et al., 1998; Nasr et al., 2019). These data suggest that the balance of GliA to GliR is critical for normal tracheal development.

Indeed, Pallister–Hall Syndrome (PHS) [Online Mendelian Inheritance of Man (OMIM): 146510] patients have a heterozygous mutation in *GLI3* that leads to a truncated protein lacking the transcriptional activation domain. As a result, the mutant protein only has GLI3R transcriptional repression even in the presence of active HH signaling. PHS patients can exhibit multiple syndromic phenotypes and often present with laryngeal clefts and tracheomalacia (Bose et al., 2002; Johnston et al., 2005).

Thus, although both HH and Wnt are critical for tracheal development, how they functionally interact is unclear. Here, we use conditional *Smo^f/f^* mouse mutants, which lack GliA, and *Gli3T^Flag/+^* transgenic mice, which overexpress Gli3R, to show that imbalance of Gli activator and repressor activity disrupts specification of Sox9^+^ tracheal chondrocytes, resulting in a tracheomalacia phenotype. We find that HH/Gli promotes the expression of Foxf1 in the ventral foregut mesenchyme, which in turn is required for Sox9 expression. Transcriptional profiling of *Foxg1Cre;Gli3T^Flag/+^* foregut tissue reveals that, in addition to loss of *Foxf1* and *Sox9*, there is a dramatic reduction in the expression of *Rspo2*, a secreted ligand known to potentiate Wnt signaling, which is required for cartilage development (Bell et al., 2008). *In situ* hybridization confirmed reduced expression of *Rspo2*, as well as the Wnt response gene *Notum* in the ventral tracheal mesenchyme (Gerhardt et al., 2018). Re-analysis of published ChIP-seq data suggests that *Rspo2* is a direct transcriptional target of Foxf1. These data reveal an HH-Foxf1-Rspo2 axis in which epithelial HH regulates Wnt signaling in the mesenchyme, promoting the specification of Sox9^+^ tracheal chondrocytes.

## RESULTS

### Tracheal chondrocytes arise from the splanchnic foregut mesoderm

In order to investigate the mechanisms of early tracheal chondrogenesis, we first performed lineage-tracing experiments to confirm that the Sox9^+^ tracheal chondrocytes are derived from the lateral plate mesoderm and not the neural crest, which give rise to laryngeal cartilage (Tabler et al., 2017). For these experiments we crossed floxed *mT/mG* reporter mice to three different Cre lines: *Foxg1Cre* which recombines in the foregut mesendoderm beginning at E8.5; *Dermo1Cre*, which recombines in the lateral plate mesoderm beginning at E9.5 (Fig. S1); or *Wnt1Cre*, which recombines in the early neural crest cells (Boucherat et al., 2015; Hébert and McConnell, 2000; Lewis et al., 2013; Li et al., 2008; Muzumdar et al., 2007; Ustiyan et al., 2018). At E13.5, the *Foxg1Cre*- and *Dermo1Cre*-expressing splanchnic mesoderm lineage traced Sox9^+^ tracheal chondrocytes surrounding the trachea, as well as the Foxf1^+^ mesenchyme and smooth muscle of the esophagus and dorsal trachealis muscle, but they did not trace the Sox9^+^ cells between the smooth muscle layers of the esophagus (Fig. S1C, Fig S2A-C). In contrast, the Wnt1^+^ cells did not trace the Sox9^+^ tracheal chondrocytes or Foxf1^+^ smooth muscle (Fig. S2B,C), but did lineage trace the Sox9^+^ enteric neurons between the esophageal smooth muscle layers (Fig. S2D), as well as Sox9^+^ chondrocytes in more anterior sections through the larynx (data not shown), consistent with previous reports (Adachi et al., 2020; Tabler et al., 2017). This demonstrates that the laryngeal and tracheal cartilages have distinct origins, with the latter arising from the lateral plate mesoderm.

### HH/Gli imbalance leads to tracheomalacia

PHS patients, with a mutated copy of *GLI3* that leads to excessive *GLI3R*, frequently present with tracheomalacia (Bose et al., 2002; Johnston et al., 2005). To better understand how disrupted HH/Gli signaling results in tracheomalacia, we analyzed a series of conditional mouse mutants in which we either deleted the HH receptor *Smo*, which effectively removes GliA, or we ectopically expressed *Gli3T^Flag/+^*, which, like PHS patients, has elevated Gli3R activity but preserved GliA function (Vokes et al., 2008)*.* We also took advantage of the different times of *Foxg1Cre* and *Dermo1Cre* recombination to examine the temporal roles of HH/Gli activity.

**Fig. 1.**
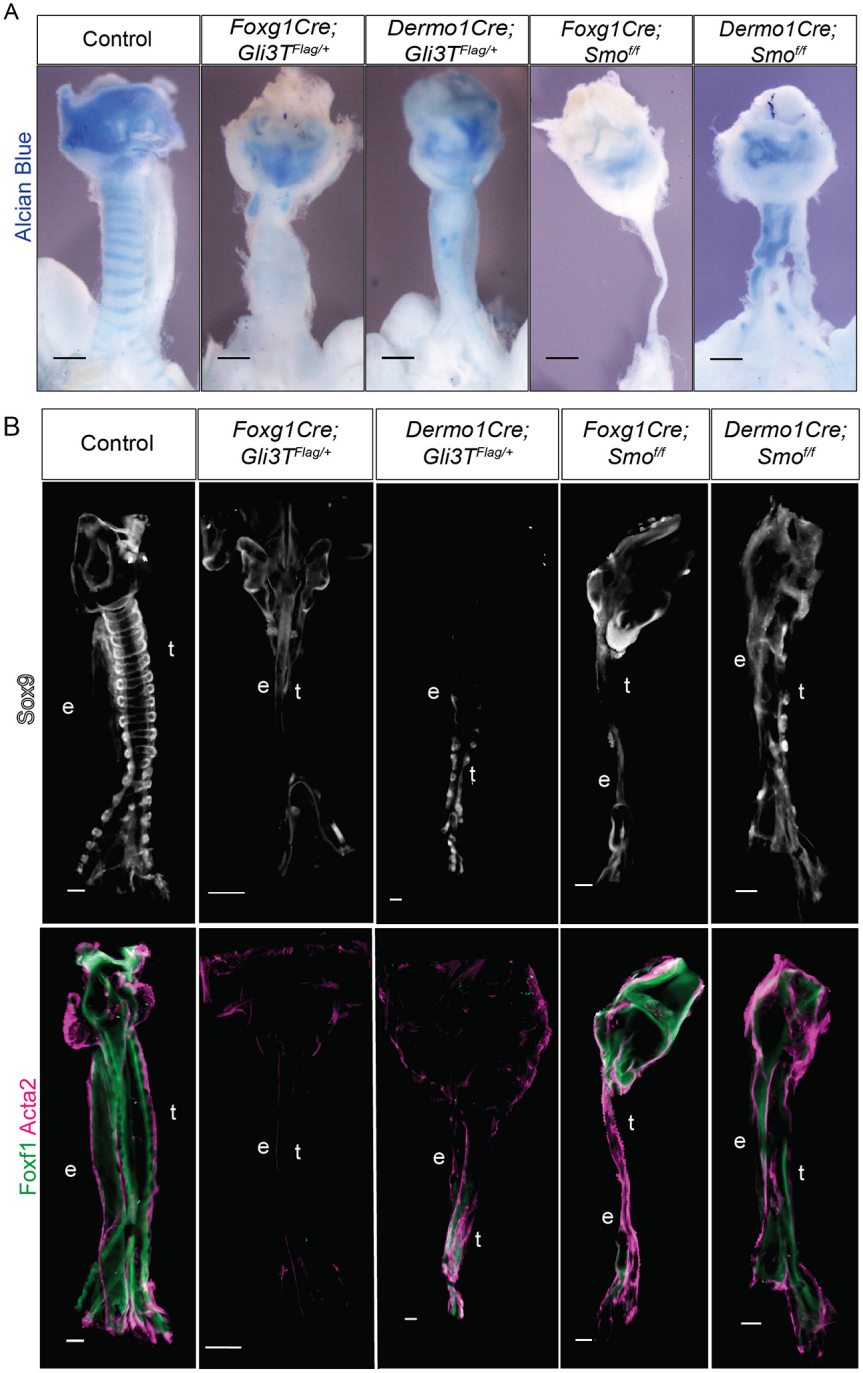
**Imbalance in Gli activity leads to tracheomalacia.** (A) Alcian Blue whole mounts of dissected E15.5 foreguts from control, *Foxg1Gli3T^Flag/+^*, *Dermo1Cre;Gli3T^Flag/+^*, *Foxg1Cre;Smo^f/f^* and *Dermo1Cre;Smo^f/f^* embryos. Earlier mutations generated using *Foxg1Cre* produced more severe tracheomalacia compared to *Dermo1Cre*-mediated deletions. *N*=3-5 embryos/genotype. (B) Sox9, Foxf1 and Acta2 whole-mount immunostaining of dissected E15.5 foreguts from control, *Foxg1Gli3T^Flag/+^*, *Dermo1Cre;Gli3T^Flag/+^*, *Foxg1Cre;Smo^f/f^* and *Dermo1Cre;Smo^f/f^* embryos. *Foxg1Cre* mutants display more significant reductions in Sox9 and Foxf1 compared to *Dermo1Cre* mutants, suggesting that impaired tracheal mesenchymal specification may contribute to tracheomalacia. *n*=3-5 embryos/genotype. Scale bars: 100 µm. e, esophagus; t, trachea.

At E15.5, all the *Gli3T^Flag/+^* and *Smo^f/f^* mutants showed varying degrees of tracheomalacia, with reduced cartilage development, as indicated by Alcian Blue staining (Fig. 1A). The early *Foxg1Cre* mutants were more severe than the later *Dermo1Cre* mutants. *Foxg1Cre;Smo^f/f^* mutants had the most severe tracheomalacia, as well as tracheal stenosis and a hypoplastic foregut, whereas the later-acting *Dermo1Cre;Smo^f/f^* mutant tracheas had relatively more cartilage than the other mutants. All mutants also showed varying losses of the dorsal trachealis muscle (Fig. 1B), as well as some degrees of esophageal stenosis, supporting that HH/Gli signaling is also required for esophageal development (Jia et al., 2018; Litingtung et al., 1998).

We next investigated whether Sox9^+^ tracheal chondrocytes were present at E15.5 but undifferentiated as a result of disrupting the HH/Gli pathway. However, all mutants showed reduced Sox9 levels that correlated with the level of Alcian Blue staining (Fig. 1B), suggesting the loss of cartilage was not due primarily to a failure in differentiation, but rather due to a loss of Sox9^+^ chondrocytes. We also observed a reduction in Foxf1, a direct Gli target that is required for foregut smooth muscle development (Hoffmann et al., 2014; Hoggatt et al., 2013; Ustiyan et al., 2018). Co-staining with Acta2 confirmed reduced smooth muscle differentiation, particularly in the *Gli3T* mutants (Fig. 1B). The more dramatic loss of Sox9^+^ tracheal chondrocytes and Foxf1^+^ muscle in *Foxg1Cre* mutants compared to *Dermo1Cre* mutants correlates with the more efficient early recombination by *Foxg1Cre* at E9.5. *Dermo1Cre* is less efficient and does not recombine robustly until E10.5 (Fig. S1A,B) (Boucherat et al., 2015; Ustiyan et al., 2018). This suggests that HH/Gli signaling begins acting in the foregut lateral plate mesoderm between E8.5 and E9.5, consistent with previous reports (Rankin et al., 2016).

### Dynamic Foxf1 and Sox9 localization during tracheal development

**Fig. 2.**
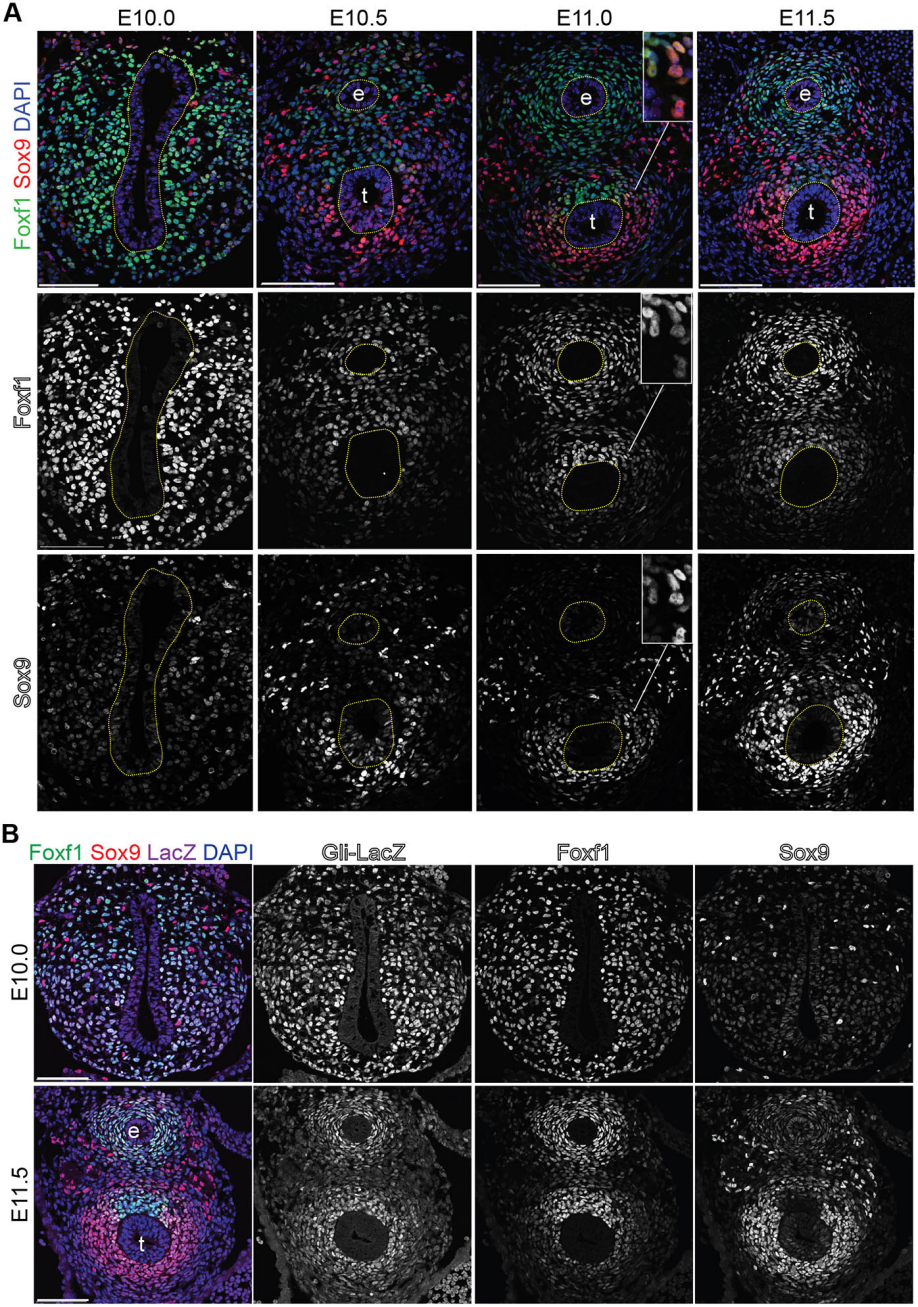
**Dynamic Sox9 and Foxf1 expression during tracheal chondrocyte specification.** (A) Foxf1 (green) and Sox9 (red) immunostaining of control embryos between E10 and E11.5. Foxf1 is initially expressed throughout the lateral plate mesoderm but then downregulated in the ventral tracheal mesoderm relative to the dorsal tracheal mesoderm by E11.5. Sox9 is only found in neural crest cells at E10 but progressively localized to the ventral tracheal mesoderm between E10.5 and E11.5. The inset shows Sox9^+^/Foxf1^+^ co-expressing cells at the boundary between the presumptive smooth muscle and chondrocytes. The dotted-yellow lines indicate the epithelial-lined trachea and esophagus lumen. *N*=3-5 embryos/stage. (B) Immunostaining of LacZ (β-galactosidase), Foxf1 and Sox9 in E10 and E11.5 Gli1^LacZ/+^ embryos, showing the direct HH-target Gli1 expressed throughout the mesoderm surrounding the esophageal and tracheal endoderm. Scale bars: 100 µm. e, esophagus; t, trachea.

As the phenotypes suggested an early disruption in chondrocyte development, we set out to better characterize the earliest expression of Sox9. Immunostaining showed that at E10, before separation of the foregut into distinct trachea and esophagus, the splanchnic mesoderm uniformly expressed Foxf1 with only rare interspersed Sox9^+^ cells (Fig. 2A). Robust Sox9 was first detected in the ventral-lateral mesoderm surrounding the trachea at E10.5 just after foregut separation, with the staining intensity and number of Sox9^+^ cells increasing by E11.5 (Fig. 2A; Hines et al., 2013). *Foxg1Cre;mTmG* lineage-tracing experiments indicated that the Sox9^+^ cells surrounding the ventral-lateral trachea were mesoderm-derived chondrocytes, whereas the dispersed Sox9^+^ cells around the presumptive esophagus were neural crest cells that gave rise to Tubb3^+^ enteric neurons (Fig. S1C). Initially Sox9 and Foxf1 were co-expressed in the ventral mesoderm, but as development proceeds, the upregulation of Sox9 in chondrocytes was coincident with a downregulation of Foxf1. By E11.5, the Sox9 and Foxf1 expression domains were largely distinct, with Foxf1 being restricted to the presumptive trachealis muscle, indicating a segregation of chondrocyte and smooth muscle lineages (Hines et al., 2013). Interestingly, Sox9/Foxf1 double-positive cells persisted at the cartilage-smooth muscle boundary (Fig. 2A, E11 inset).

As upregulation of Sox9 and downregulation of Foxf1 in the ventral tracheal mesenchyme follows tracheoesophageal separation, we investigated whether these expression dynamics were dependent on tracheoesophageal separation and/or epithelial identity. Nkx2-1 and Sox2 are transcription factors required for the development of the tracheal and esophageal endoderm epithelia, respectively (Minoo et al., 1999; Que et al., 2007). *Nkx2-1^−/−^* mutants have a single undivided foregut tube of esophageal character, whereas deletion of *Sox2* from the foregut results in an undivided foregut tube of tracheal character (Kuwahara et al., 2020; Que et al., 2009, 2007; Teramoto et al., 2020; Trisno et al., 2018). A re-analysis of these mutants showed that the single undivided foregut in both the *Sox2* and *Nkx2-1* mutant embryos was correctly patterned. However in *Nkx2-1* mutants there appeared to be fewer Sox9^+^ chondrocytes compared to controls or *Sox2* mutants, whereas the *Sox2* mutants seemed to have far fewer Foxf1^+^ cells compared to controls or *Nkx2-1* mutants (Fig. S3). Thus, the emergence of tracheal chondrocytes with an upregulation of Sox9 and a downregulation of Foxf1 is influenced by the epithelial identity but not dependent on tracheoesophageal separation.

### Hedgehog/Gli activity is required for specification of the tracheal mesenchyme

**Fig. 3.**
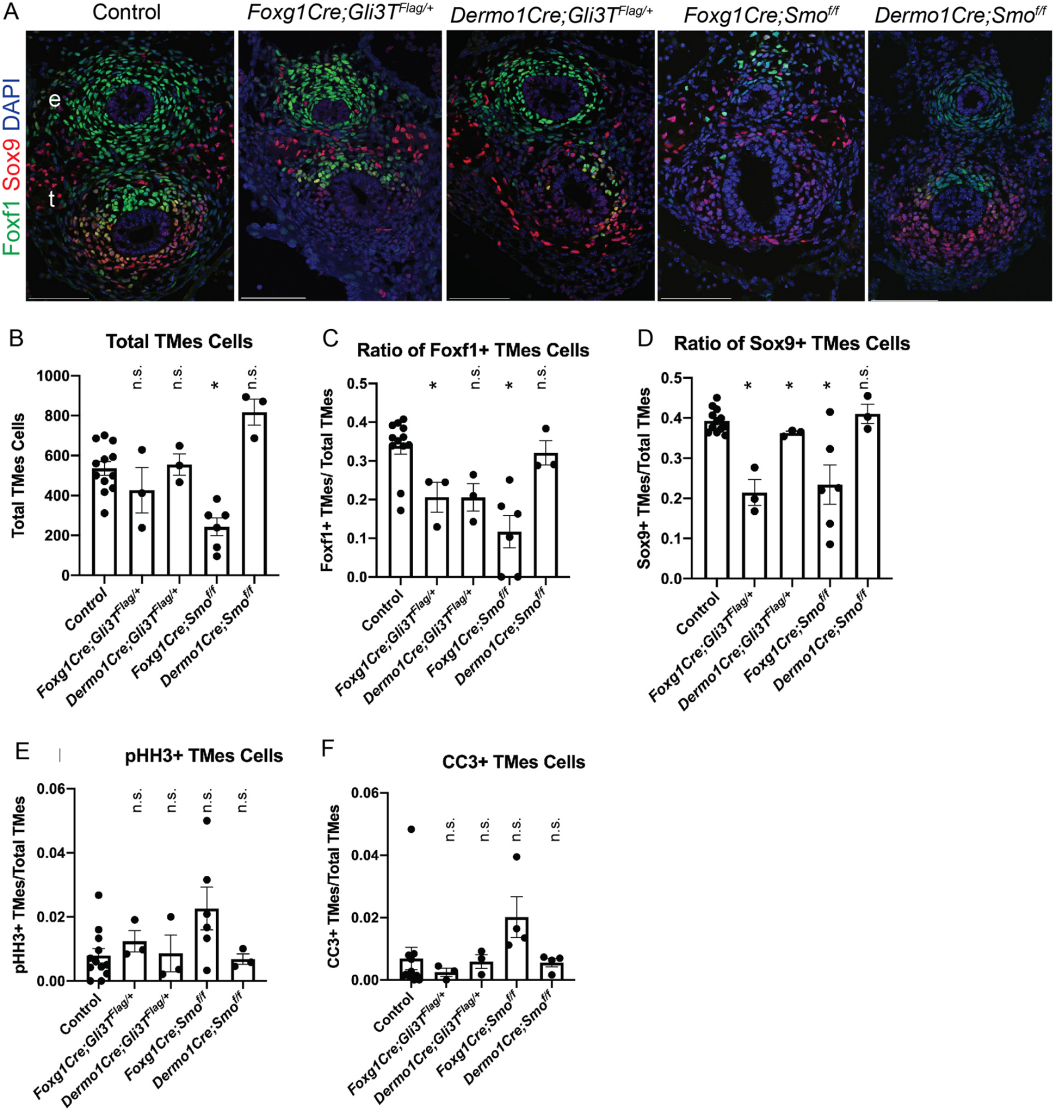
**Hedgehog/Gli signaling supports tracheal mesenchymal specification.** (A) Foxf1 (green) and Sox9 (red) immunostaining of E11.5 foregut transverse sections from control, *Foxg1Cre;Gli3T^Flag/+^*, *Dermo1Cre;Gli3T^Flag/+^*, *Foxg1Cre;Smo^f/f^* and *Dermo1Cre;Smo^f/f^* embryos. *Foxg1Cre* mutants have fewer Foxf1^+^ and Sox9^+^ mesoderm cells compared to *Dermo1Cre* mutants and control embryos. *N*=3-5 embryos/genotype. Scale bars: 100 µm. e, esophagus; t, trachea. (B-F) Quantification of E11.5 immunostaining for each genotype showing the total number of cells in the tracheal mesoderm (B, TMes), the ratio of Foxf1^+^/total TMes cells (C), the ratio of Sox9^+^/total TMes cells (D), the mitotic index of phospho-histone H3 (pHH3^+^)/total TMes cells to indicate proliferation (E), and the ratio of cleaved caspase-3 (CC3^+^)/total TMes cells to indicate apoptosis (F). Histograms are mean±s.e.m., with data points for each individual embryo shown. *P<0.05 (unpaired two-sided Student's *t*-test with unequal variance). n.s., not significant. *N*=3-5 embryos/genotype.

We next considered whether dynamic HH signaling might account for the reciprocal Sox9-Foxf1 expression pattern. Analysis of *Shh^GFP/+^* embryos with GFP knocked into the *Shh* locus, as well as RNAScope *in situ* hybridization, showed that at E10.5, *Shh* was enriched in the tracheal epithelium, but by E11.5, *Shh* was more strongly expressed in the esophageal epithelium (Fig. S4), consistent with previous reports (Ioannides et al., 2003). In contrast *Ihh* was weakly expressed in the E10.5 trachea epithelium and mesenchyme but undetectable by E11.5 (Fig. S4B). We postulated that this expression pattern might result in an overall reduction of HH response in the ventral tracheal mesoderm correlating with reduced Foxf1 and increased Sox9. We took advantage of *Gli1LacZ* reporter mice as Gli1 is a direct transcriptional target of HH-Gli2/3 signaling, enabling us to examine the overall impact of both Shh and Ihh activity (Briscoe and Thérond, 2013). Contrary to our hypothesis, Gli1LacZ was uniformly expressed in the foregut mesoderm surrounding the gut tube at both E10 and E11, with no obvious difference in trachea versus esophageal mesenchyme (Fig. 3B). RNAScope *in situ* hybridization confirmed this *Gli1* expression pattern and also showed uniform *Smo* expression in the foregut, supporting the conclusion that HH/Gli signaling is still active in the E11.5 ventral tracheal mesoderm (Fig. S4C).

Next, we performed Foxf1 and Sox9 immunostaining on *Gli3T^Flag/+^* and *Smo^f/f^* mutants at E11.5 to examine the initial defects in tracheal chondrogenesis (Fig. 3A). The *Foxg1Cre;Smo^f/f^* and *Foxg1Cre;Gli3T^Flag/+^* mutants had a reduced number of Sox9^+^ chondrocytes and also exhibited reduced ventral Foxf1 expression compared to controls (Fig. 3A-D). *Dermo1Cre* mutants appeared to be mostly unchanged in both overall tracheal mesoderm cell number and lineage-specific populations, although they did exhibit a trend of reduced Foxf1 expression levels (Fig. 3A-C). As we observed loss of Sox9 and Foxf1 at E15.5 in both *Dermo1Cre* mutants, this suggested a continuing role for HH/Gli signaling in maintaining Foxf1 and Sox9 expression and promoting tracheal chondrogenesis.

As HH signaling is known to maintain cell proliferation and survival in many contexts (Bohnenpoll et al., 2017; Li et al., 2004), we assessed whether this might contribute to the tracheomalacia phenotype in *Smo^f/f^* and *Gli3T^Flag/+^* mutants. At E11.5, there were no statistically significant changes in either tracheal mesodermal cell proliferation or apoptosis in any of the mutants as determined by quantification of phospho-histone H3 or cleaved caspase-3 immunostaining, respectively (Fig. 3E,F; Fig. S5A). However, previous studies have demonstrated that HH/Gli does indeed promote splanchnic mesoderm proliferation and survival from E8.5 to E9.5, which likely explains the reduced cell number in *Foxg1Cre* mutants, which recombines starting at E8.5 (Fig. 3B; Li et al., 2008; Rankin et al., 2016). However, overall, the reduced cell numbers in the *Foxg1Cre* mutant is not sufficient to explain the loss of Foxf1 and Sox9. Together, the results indicate that HH/Gli is required between E8.5 and E10.5 to maintain Foxf1 and specify Sox9^+^ chondrocytes, with prolonged signaling between E10.5 and E15.5 maintaining cartilage and smooth muscle development.

### Foxf1 is required for development of Sox9^+^ chondrocytes

**Fig. 4.**
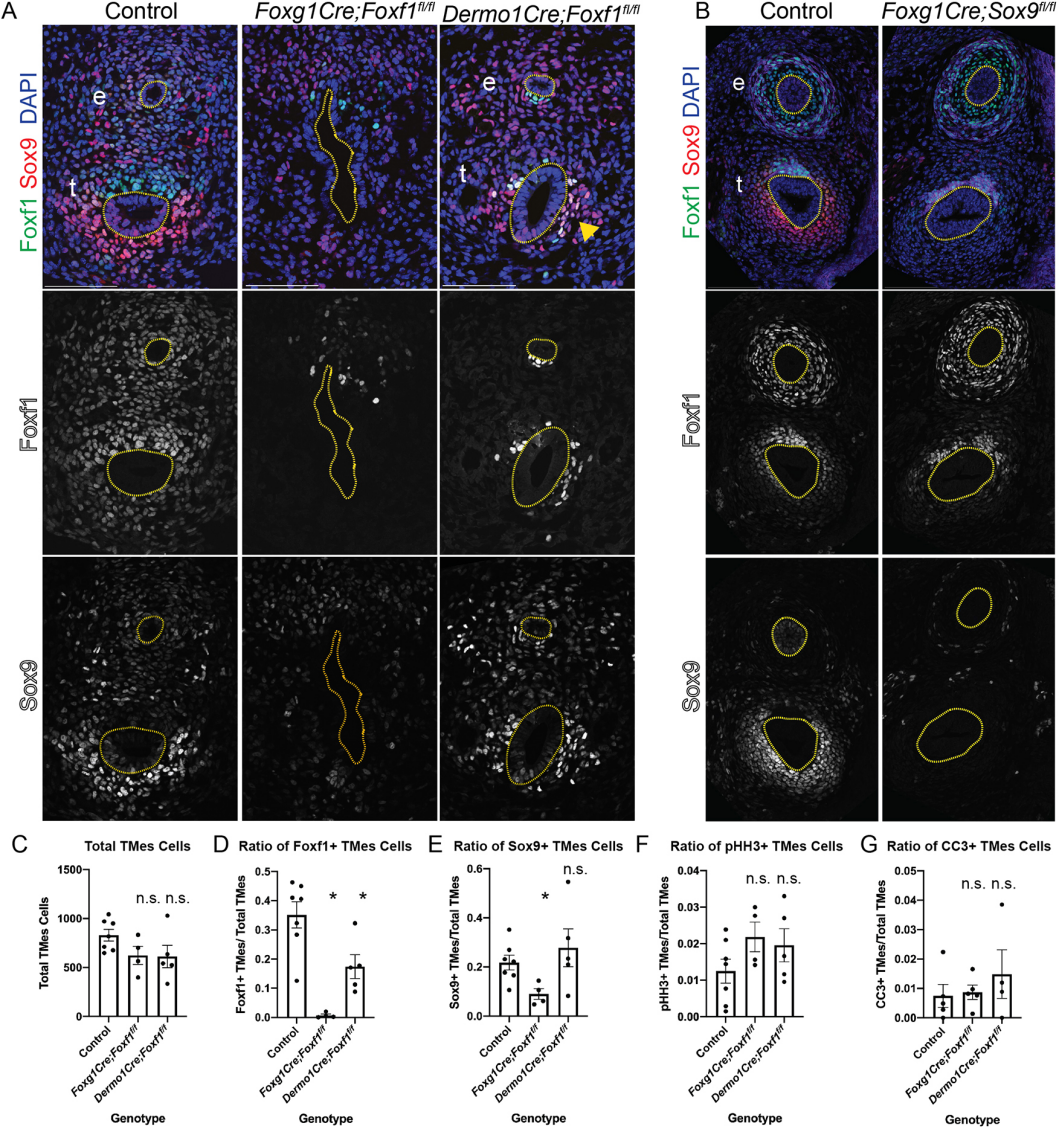
**Foxf1 is required for tracheal Sox9 expression.** (A) Foxf1 (green) and Sox9 (red) immunostaining of E11.5 control, *Foxg1Cre;Foxf1^f/f^* and *Dermo1Cre;Foxf1^f/f^* mutants. *Foxg1Cre* mutants have fewer Foxf1^+^ and Sox9^+^ cells compared to controls. *Dermo1Cre* mutants have some Sox9^+^ cells in the ventral trachea that co-localize with Foxf1 (arrowhead), suggesting that these cells escaped Cre recombination. (B) Foxf1 and Sox9 immunostaining of E13.5 control and *Foxg1Cre;Sox9^f/f^* embryos. Dotted lines indicate the trachea and esophagus. (C-G) Quantification of E11.5 immunostaining for each genotype showing the total number of cells in the tracheal mesoderm (C, TMes), the ratio of Foxf1^+^/total TMes cells (D), the ratio of Sox9^+^/total TMes cells (E), the mitotic index of phospho-histone H3 (pHH3^+^)/total TMes cells to indicate proliferation (F), and the ratio of cleaved caspase-3 (CC3^+^)/total TMes cells to indicate apoptosis (G). Histograms are mean±s.e.m., with data points for each individual embryo shown. *P<0.05 (unpaired two-sided Student's *t*-test with unequal variance). n.s., not significant. *N*=3-5 embryos/genotype. Scale bars: 100 µm. e, esophagus; t, trachea.

Previous work has shown that loss of one *Foxf1* allele leads to impaired tracheal and esophageal development (Mahlapuu et al., 2001; Ustiyan et al., 2018). The reciprocal expression pattern of Foxf1 and Sox9, and the fact that both are reduced in the *Smo^f/f^* and *Gli3T^Flag/+^* mutants, suggest that Foxf1 may initially be required for the development of Sox9^+^ progenitors, but that Sox9 and Foxf1 might then antagonize the expression of each other. To test this, we conditionally deleted *Foxf1* using both *Foxg1Cre* and *Dermo1Cre*. *Foxg1Cre;Foxf1^f/f^* mutants exhibited a large reduction in Sox9^+^ ventral foregut mesoderm cells (Fig. 4A), consistent with the small cartilaginous nodules previously observed in *Foxf1^+/−^* tracheas (Mahlapuu et al., 2001). *Dermo1Cre;Foxf1^f/f^* mutants also exhibited fewer Sox9^+^ cells compared to controls (Fig. 4A). Quantification of cell numbers revealed that at E11.5, *Foxf1* mutants had a general trend of ∼200 fewer tracheal mesoderm cells compared to controls, whereas only the *Foxg1Cre;Foxf1^f/f^* mutants had significantly fewer Sox9^+^ cells in the tracheal mesoderm (Fig. 4C-E). Interestingly, some of the remaining Sox9^+^ cells also expressed Foxf1, suggesting that they escaped Cre recombination (Fig. 4A; arrowhead ). The fact that Sox9 was not upregulated in the *Dermo1Cre;Foxf1^f/f^* mutants indicates that Foxf1 does not repress Sox9, which was one possibility suggested by their reciprocal expression patterns. We also found that the *Foxg1Cre;Foxf1^f/f^* mutant foregut failed to separate into a distinct trachea and esophagus (Fig. 4A), consistent with our recent work suggesting that the early lateral plate mesoderm is required for morphogenesis (Nasr et al., 2019).

We next examined whether Sox9 might suppress the ventral expression of Foxf1 as chondrocytes emerge. Examination of E13.5 *Foxg1Cre;Sox9^f/f^* mutants revealed that loss of Sox9 had no impact on the ventral downregulation of Foxf1 (Fig. 4B). This suggests that Sox9 and its downstream targets are not responsible for the ventral reduction in Foxf1 expression. Thus, Foxf1 and Sox9 do not repress the expression of each other during tracheal chondrogenesis.

As previous work showed that few *Dermo1Cre;Foxf1^f/f^* mutants survive beyond E16.5 due to impaired growth and survival of the cardiovascular and pulmonary mesenchyme (Ustiyan et al., 2018), we examined cell proliferation and apoptosis. However, at E11.5, we did not detect any significant differences in cell proliferation or cell apoptosis in either *Foxg1Cre;Foxf1^f/f^* or *Dermo1Cre;Foxf1^f/f^* compared to controls (Fig. 4F,G; Fig. S5B). As neither changes in cell proliferation nor cell death can explain the relative reduction in Sox9^+^ cells in *Foxg1Cre;Foxf1^f/f^* mutants, we conclude that Foxf1 is required for initial specification of Sox9^+^ tracheal chondrocyte.

### Gli3 and Foxf1 regulate expression of *Rspo2*, a known Wnt modulator of tracheal chondrogenesis

**Fig. 5.**
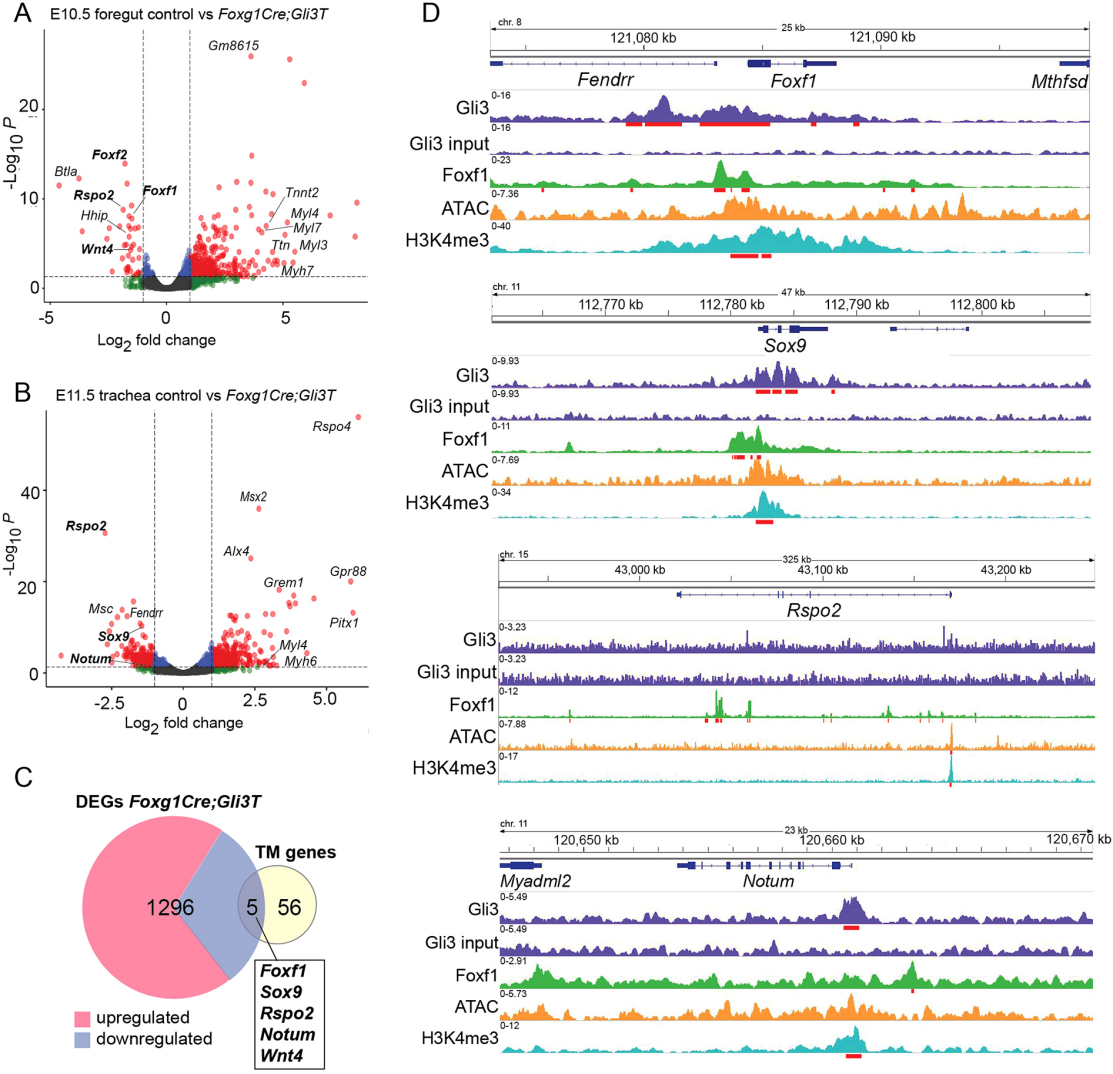
**Gli3 regulates expression of Wnt pathway components.** (A,B) Volcano plot of differentially expressed transcripts in control versus *Foxg1Gli3T^Flag/+^* foreguts, as determined by Log_2_FC≥|1| (*P*<0.05) at E10.5 (A) and E11.5 (B). Dashed lines indicate FC and P value thresholds. (C) Venn diagram intersecting genes differentially expressed in *Foxg1Cre;Gli3T^Flag/+^* mutants with genes known to be involved in human or mouse tracheal chondrogenesis (Shefchek et al., 2020; Sinner et al., 2019). TM, tracheomalacia-associated genes. (D) Genome browser views of Gli3-3xFlag (GSE133710), Foxf1 (GSE77159) and H3K4me3 (GSE119885) ChiP-seq data, as well as ATAC-seq data (GSE119885) on *Foxf1*, *Sox9*, *Rspo2* and *Notum* loci. Gli3 and Foxf1 bind the *Foxf1*, *Sox9* and *Notum* loci, but only Foxf1 shows direct binding of the *Rspo2* locus along with significant ATAC and H3K4me3 peaks, suggesting active transcription. Statistically significant ChIP peaks are underlined in red.

Previous studies have shown, in different cellular contexts, that both *Foxf1* and *Sox9* are direct transcriptional targets of HH/Gli (Bien-Willner et al., 2007; Hoffmann et al., 2014; Madison et al., 2009; Tan et al., 2018). In order to discover additional Gli-regulated genes that might mediate tracheal chondrogenesis, we performed RNA-seq on E10.5 foreguts and E11.5 tracheas dissected from control and *Foxg1Cre;Gli3T^Flag/+^* embryos. Differential expression analysis (Log_2_ Fold Change ≥|1|, *P*<0.05) identified 708 transcripts (70 reduced and 638 increased) with altered expression in mutants at E10.5, and 738 Gli-regulated transcripts (352 reduced and 386 increased) at E11.5 (Fig. 5A,B; Table S1). Of these, 144 genes were differentially expressed at both E10.5 and E11.5. The reduced expression of *Hhip*, a direct HH target gene, was consistent with Gli3T repressive activity (Beachy et al., 2010). Gene ontology enrichment analysis of the downregulated genes identified epithelial tube morphogenesis and respiratory system development, consistent with HH signaling being required for foregut organogenesis, whereas upregulated genes were associated with cell signaling and muscle development, indicative of the relative increase in muscle progenitors in the absence of Sox9^+^ chondrocytes (Fig. S6A,B).

We next intersected the Gli3R-regulated transcripts with a manually curated list of 61 genes implicated in tracheal chondrogenesis and/or tracheomalacia in mice or humans (Table S2). These were identified from a review of the literature (Sinner et al., 2019) and by searching the Monarch Initiative (https://monarchinitiative.org/), an online knowledgebase that aggregates human disease and animal model genotype-phenotype associations (Shefchek et al., 2020). This intersection revealed five genes, all of which were downregulated in Gli3T transgenic embryos (Fig. 5C). In addition to *Sox9* and *Foxf1*, this identified *Rspo2*, *Wnt4* and *Notum*, all key regulators of the canonical Wnt pathway (Fig. 5A-C) and all of which exhibit impaired tracheal chondrogenesis when mutated in mice (Bell et al., 2008; Caprioli et al., 2015; Gerhardt et al., 2018). Focusing on the Wnt pathway, we additionally observed reduced expression of *Wnt11* (Fig. S6C), the role of which in tracheal development has not yet been identified but is known to support Sox9^+^ chondrocyte maturation in other tissues (Liu et al., 2014; Tada and Smith, 2000). *Rspo2*, a secreted protein that interacts with Lgr4/5/6 and Lrp6 receptor complexes to potentiate Wnt/β-catenin signaling, was one of the most downregulated transcripts at both E10.5 and 11.5 (−1.86 and −2.73 Log_2_FC, respectively) (Bell et al., 2008; Carmon et al., 2011; de Lau et al., 2011; Gong et al., 2012; Kazanskaya et al., 2004; Kim et al., 2008; Lebensohn and Rohatgi, 2018; Ruffner et al., 2012). *Wnt4* was modestly downregulated in the E10.5 foregut (−1.54 Log_2_FC), whereas Notum, a known Wnt target gene and feedback inhibitor was reduced about twofold in the E11.5 Gli3T trachea (Fig. 5B,C; Fig. S6C; Gerhardt et al., 2018). These data demonstrate that HH/Gli transcriptionally regulates components of the canonical Wnt pathway, which are known to activate Sox9 expression in the tracheal mesenchyme (Snowball et al., 2015).

We next examined published ChIP-seq data to examine whether *Rspo2*, *Notum*, *Wnt4* and *Wnt11* were likely to be direct target genes of Gli and Foxf1 transcription factors. We used previously published Gli3-3xFlag ChIP from E10.5 limb buds and Foxf1 ChIP from E18.5 lungs (Dharmadhikari et al., 2016; Lex et al., 2020); these datasets are the most similar to tracheal chondrocytes currently available. We also examined previously published ATAC-seq and H3K4me3 ChIP-seq performed in the E9.5 cardiopulmonary foregut progenitors to help identify active promoter and enhancer regions (Steimle et al., 2018). Examination of genome browsers showed that Gli3 can bind to both the *Foxf1* and *Sox9* promoters overlapping with H3K4me3 peaks (Fig. 5D), consistent with previous reports that they are direct HH/Gli targets (Hoffmann et al., 2014; Tan et al., 2018; Vokes et al., 2008). Gli3 binding regions were also detected on putative regulatory elements of *Notum* and *Wnt 11* but not on the *Rspo2* or *Wnt4* loci (Fig. 5D; Fig. S6F), suggesting that *Rspo2* and *Wnt4* might be indirectly regulated by Gli. Indeed, the Foxf1 ChIP-seq data showed that Foxf1 binding was associated with putative intronic enhancers of *Rspo2*; with the *Sox9*, *Notum*, *Wnt4* and *Wnt11* loci; as well as with the *Foxf1* promoter itself (Fig. 5D; Fig. S6F) (Ustiyan et al., 2018). Although the ChIP data are not from the developing trachea, together with the RNA-seq, this analysis is consistent with Gli3-Foxf1 acting in a regulatory network to promote the expression of *Sox9*, *Foxf1*, *Rspo2*, *Notum*, *Wnt4* and *Wnt11* in the presumptive chondrocytes.

### Wnt signaling is disrupted in *Gli3R* and *Foxf1* mutants

**Fig. 6.**
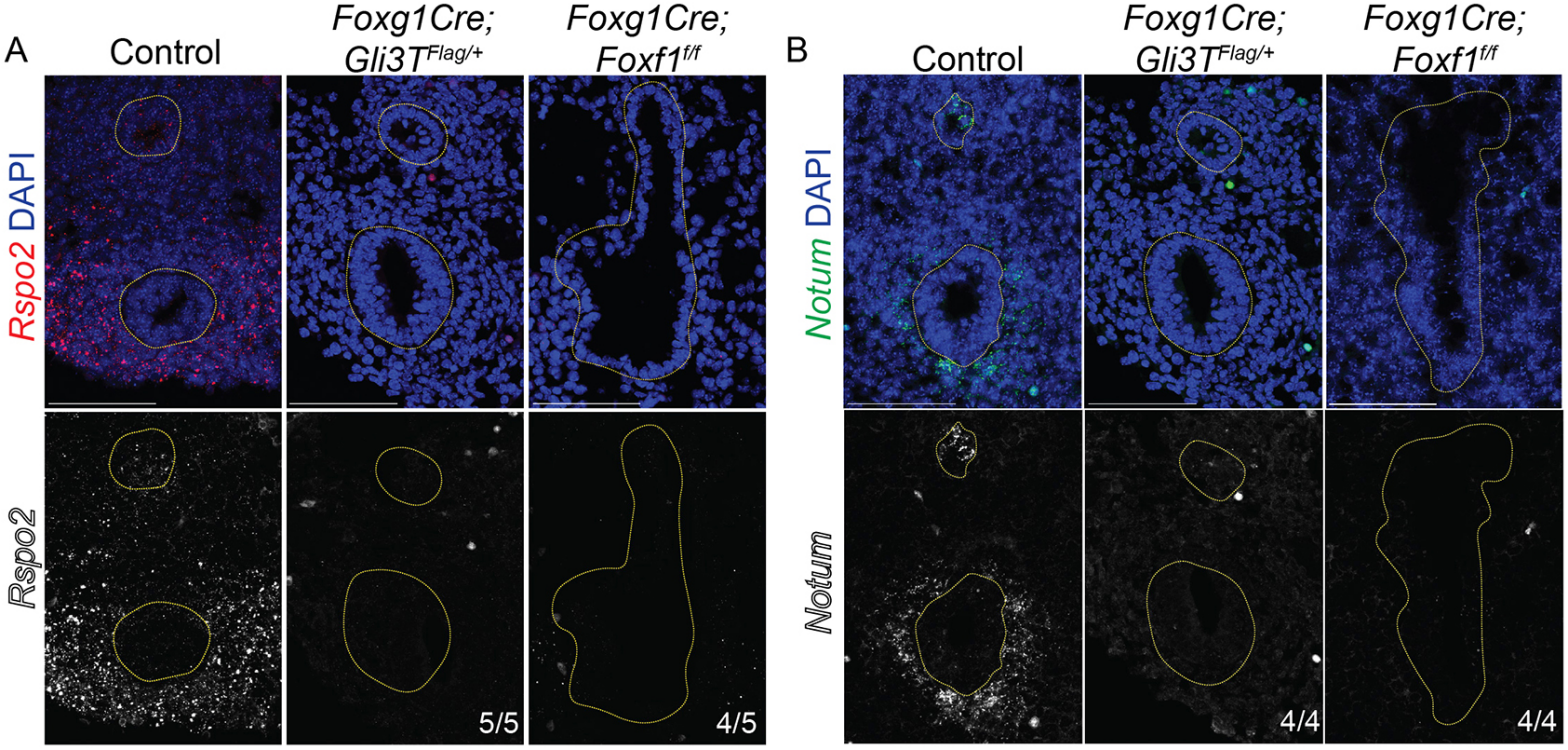
**Expression of Wnt pathway genes *Rspo2* and *Notum* are reduced in *Gli* and *Foxf1* Mutants.** (A,B) RNAscope *in situ* hybridization of E11.5 control, *Foxg1Cre;Gli3T^Flag/+^* and *Foxg1Cre;Foxf1^f/f^* embryos reveals decreases in *Rspo2* (A, red) and *Notum* (B, green) in the ventral-lateral tracheal mesenchyme. These results suggest that HH/Gli-Foxf1 signaling is upstream of *Rspo2* and *Notum* during tracheal development. Yellow-dotted lines outline the epithelia of the trachea and esophagus lumen. Scale bars: 100 µm. e, esophagus; t, trachea.

We performed RNAscope *in situ* hybridization on E11.5 embryos to validate the RNA-seq analysis and examine which cell populations exhibited a change in *Rspo2*, *Notum* and *Wnt4* expression. In controls, *Rspo2* and *Notum* were strongly expressed in the ventral tracheal mesoderm, whereas *Wnt4* was weakly expressed in the mesoderm surrounding the esophagus and trachea, as well as in the epithelium (Fig. 6A,B; Fig. S6E), all consistent with previous publications (Bell et al., 2008; Caprioli et al., 2015; Gerhardt et al., 2018). In both *Foxg1Cre;Gli3T^Flag/+^* and *Foxg1Cre;Foxf1^f/f^* mutants, *Rspo2* and *Notum* were largely undetectable relative to controls, and there was a modest reduction of *Wnt4* levels (Fig. 6A,B; Fig. S4E). As *Notum* is a direct Wnt target gene required for tracheal chondrogenesis (Gerhardt et al., 2018), this suggests that the cumulative reduction in Rspo2, Wnt4 and Wnt11 in the ventral mesenchyme of *Foxg1Cre;Gli3T^Flag/+^* and *Foxg1Cre;Foxf1^f/f^* mutants results in an overall reduction in Wnt response that is unable to sustain Sox9 induction. Together, these data demonstrate that HH/Gli regulate a Foxf1-Wnt pathway required for tracheal chondrogenesis.

## DISCUSSION

**Fig. 7.**
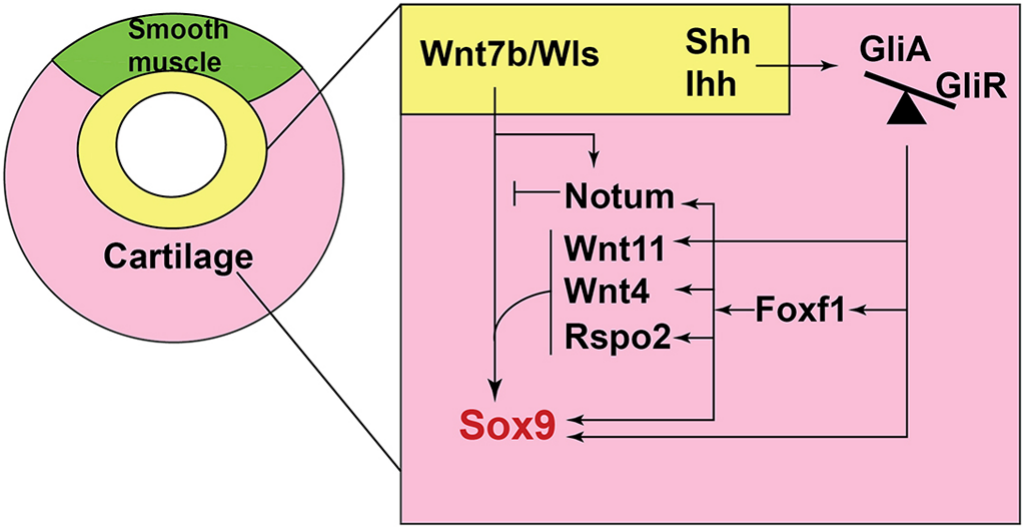
**Model of a HH/Gli-Foxf1-Wnt signaling network controlling Sox9^+^** **chondrogenesis.** HH/Gli signals from the epithelium (yellow) result in more Gli2/3 activator (GliA) than Gli repressor (GliR). Activated Gli2/3 directly stimulates *Foxf1* expression, which in turn supports growth and survival of the tracheal mesoderm (pink). Foxf1 and Gli transcription factors cooperate to directly promote Sox9 transcription. In addition, Gli and Foxf1 promote the expression of a number of Wnt pathway components in the mesenchyme, including *Rspo2*, *Wnt4*, *Wnt11* and *Notum*, which act in concert with epithelial *Wnt7b* and *Wls* to further enhance and maintain *Sox9* expression. Wnt/β-catenin signaling in the ventral tracheal mesoderm is essential for tracheal chondrogenesis. Notum, another direct Gli and Foxf1 target, attenuates Wnt/β-catenin signaling, possibly to regulate chondrocyte maturation. In the dorsal tracheal mesenchyme (green), which is thought to have lower Wnt and BMP signaling, Gli-Foxf1 does not activate Sox9^+^ chondrogenesis, and the tissue adopts a smooth muscle fate.

In this study, we show that conditional mouse mutants with relatively high levels of GliR, mimicking PHS, exhibit tracheomalacia and fail to properly specify Sox9^+^ tracheal chondrocytes. Our data suggest a model of the epithelial-mesenchymal interactions that orchestrate tracheal chondrocyte differentiation (Fig. 7) in which: (1) HH ligands expressed in the ventral foregut epithelium from E8.5 to E11.5 signal to the surrounding splanchnic mesoderm to activate Gli transcription factors that promote *Foxf1* transcription; (2) Foxf1 in turn maintains the lateral plate mesoderm and directly promotes *Sox9* transcription at the initiation of tracheal chondrogenesis; (3) downstream of HH, Gli3 and Foxf1 cooperate in a regulatory network to promote the transcription of *Sox9*, *Rspo2*, *Wnt4*, *Wnt11* and *Notum*; and (5) this Gli-Foxf1-Rspo2 axis promotes Wnt signaling in the mesenchyme, which is known to be required for the activation of *Sox9* expression and tracheal cartilage development (Snowball et al., 2015). Disruptions in this HH-Wnt regulatory network result in the failure to induce and/or maintain Sox9, which is essential for chondrogenesis. Together, these data provide a mechanistic basis for the tracheomalacia in patients with mutations in HH/Gli pathway genes.

### Temporal role of HH/Gli in tracheal chondrogenesis

Previous work indicates that conditional deletion of *Shh* from the respiratory epithelium between E8.5 and E12.5 resulted in minor disruptions to tracheal cartilage ring patterning, whereas deletion after E13.5 had no effect (Miller et al., 2004). We postulate that the differences in this report compared to our current study may be due to a low level of Ihh acting redundantly with Shh; Ihh promotes chondrocyte proliferation in the endochondral skeleton (Long et al., 2001). We manipulated the Smo receptor or Gli3 downstream of any ligand redundancy. An alternative explanation is the efficiency of early Cre-mediated deletion. The fact that the early-acting *Foxg1Cre* had a more severe loss of Foxf1 and Sox9 than *Dermo1Cre* mutants suggests that HH starts acting on the lateral plate mesoderm between E8.5 and E10.5. However, by E15.5, the *Dermo1Cre* mutants did exhibit a dramatic loss of cartilage and reduced Sox9, suggesting that continued HH/Gli activity between E10.5 and E15.5 is necessary to maintain Sox9 and to promote chondrogenesis.

Our study also suggests that the balance of GliA to GliR activity is critical for specification of Sox9^+^ chondrocytes. Both the *Smo^f/f^* mutants, which mimic an absence of GliA function, and *Gli3T^Flag/+^* mutants, which have excess Gli3R, exhibit tracheomalacia and a reduction of Sox9. Similarly, *Gli2^−/−^;Gli3^+/−^* germline mouse mutants, with one copy of Gli3R but no Gli2A, displayed tracheomalacia, whereas *Gli2^+/−^;Gli3^−/−^* mice, which lack Gli3R, do not (Litingtung et al., 1998; Miller et al., 2004; Nasr et al., 2019; Park et al., 2010). These phenotypes, along with the genomic analysis, suggest that too much Gli3R relative to Gli2A directly represses *Sox9* transcription. Indeed, previous work indicates that HH target gene expression can be reduced by either the loss of GliA or by increased GliR relative to GliA, whereas in some cases loss of GliR is sufficient to activate some target genes (Falkenstein and Vokes, 2014).

It is also possible that spatiotemporal dynamics in HH signaling levels impact dorsal-ventral patterning of the peritracheal mesenchyme. Although we observed uniform expression of the HH target gene *Gli1* in the mesenchyme around the trachea and esophagus, it is possible that by E11.5 there is insufficient HH activity in the ventral trachea to support *Foxf1* expression. This could be a product of the shift in *Shh* expression from the ventral foregut to the esophageal epithelium. Ultimately, this might result in an HH activity gradient that patterns the peritracheal mesenchyme and could explain in part why Foxf1 persists in the dorsal trachealis muscle next to the Shh-rich esophagus.

### Foxf1 is required for specification of Sox9^+^ tracheal chondrocytes

Our analysis indicates that Foxf1 is required for specification of Sox9^+^ tracheal chondrocytes. We postulate that Foxf1 promotes Sox9 expression in several ways. First, Foxf1 is known to be essential for mesenchymal proliferation and survival of the early foregut mesenchyme (Rankin et al., 2016), and although this alone cannot account for the phenotypes we observe, we expect that it contributes to the ultimate expansion of chondrogenic mesenchyme. Second, the ChIP-seq analysis suggests that Foxf1 directly regulates *Sox9* transcription. Finally, Foxf1 also promotes *Sox9* expression indirectly by stimulating expression of Wnt pathway components.

The downregulation of Foxf1 in the ventral mesoderm as Sox9^+^ chondrocytes are induced initially suggested that Foxf1 and Sox9 might mutually repress one another, but the genetic analysis ruled out this possibility. Rather, our data together with previous studies suggest that downstream of HH/Gli and Foxf1, pathways including Wnt and possibly BMP likely contribute to regulatory feedback loops controlling dorsal-ventral patterning of the peritracheal mesoderm. This patterning likely leads to the restriction of Foxf1 to the dorsal trachealis and Sox9 to a ventral-lateral domain (Domyan et al., 2011; Rajagopal et al., 2008; Rankin et al., 2016; Snowball et al., 2015).

### HH/Gli regulates a Foxf1-Rspo2-Wnt axis

Although both HH and Wnt signaling were known to regulate tracheal chondrogenesis, how these pathways interact was previously unclear. Our analysis indicates that epithelial HH signals stimulate Gli activity in the adjacent ventral mesenchyme to activate a Foxf1-Rspo2-Wnt signaling axis that promotes Sox9 expression. During tracheal development, Wnt ligands secreted from the ventral respiratory epithelium (primarily Wnt7b) are required to signal to the adjacent mesenchyme to activate Sox9 expression (Rajagopal et al., 2008). Conditional epithelial deletion of the Wnt cargo protein *Wls*, which is required for Wnt ligand secretion, results in a failure to specify Sox9^+^ chondrocytes (Snowball et al., 2015). In addition, a number of other Wnt pathway components, including Rspo2, Wnt4, Wnt5a, Wnt2 and Notum are expressed in the peritracheal mesenchyme and contribute to development of Sox9^+^ chondrocytes (Bell et al., 2008; Caprioli et al., 2015; Gerhardt et al., 2018; Goss et al., 2009; Kishimoto et al., 2018; Li et al., 2002; Snowball et al., 2015). For example, *Rspo2* and *Wnt4* mutant tracheas have fewer and dysmorphic tracheal cartilage rings, and *Notum* mutant tracheas exhibit impaired cartilage differentiation (Bell et al., 2008; Caprioli et al., 2015; Gerhardt et al., 2018).

Our analysis suggests that *Rspo2*, *Wnt4*, *Wnt11*, *Notum* and *Sox9* are all direct Foxf1 targets, and that Gli3 might bind to the same *Sox9*, *Notum* and *Wnt4* enhancers as Foxf1. This implies a positive feedback loop in which Gli transcription factors first activate Foxf1 in the early lateral plate mesoderm. Foxf1 then cooperates with Gli to directly promote expression of *Sox9* and the Wnt pathway, which in turn reinforces *Sox9* transcription. Indeed, there is genomic evidence from the developing heart and long bones supporting such a Gli-Fox combinatorial activity (Hoffmann et al., 2014; Tan et al., 2018). One limitation of our study was that the ChIP-seq data was from other tissue. Future ChIP experiments of Gli3 and Foxf1 from the fetal trachea will be important to elucidate the genomic details of this Gli-Foxf1-Wnt regulatory network.

Our data further suggest that the combined loss of *Rspo2*, *Wnt4* and *Notum* in *Foxg1Cre;Gli3T^Flag/+^*, *Foxg1Cre;Smo^f/f^* and *Foxg1Cre;Foxf1^f/f^* mutants results in a reduction of Wnt activity insufficient to activate and/or maintain *Sox9* transcription, similar to *Wls* mutants. Indeed, the tracheomalacia observed in *Rspo2* mutants is made worse with additional reduction in *Lrp6*. This suggests that a dose-dependent disruption of Wnt activity may severely impact tracheal chondrogenesis (Bell et al., 2008).

The regulation of *Sox9* expression by Wnt signaling appears to be context dependent. Although Wnt-β-catenin promotes *Sox9* expression in the trachea and the gut, it appears to suppress *Sox9* in the context of limb chondrogenesis (Blache et al., 2004; Kozhemyakina et al., 2015; Snowball et al., 2015). The prevailing view is that canonical Wnt signaling directly regulates *Sox9* transcription, but to our knowledge, direct binding of the Wnt transcriptional effectors Tcf and β-catenin to *Sox9* enhancers remains to be demonstrated. It will be interesting to determine whether Tcf-β-catenin complexes bind to the same enhancers as Foxf1 and Gli3. Finally, it is possible that Wnt also contributes indirectly as β-catenin can promote FGF-dependent tracheal chondrogenesis (Hou et al., 2019). Altogether, our data provide a mechanistic understanding of how disruptions in HH/Gli signaling may impair specification of Sox9^+^ tracheal chondrocytes and ultimately lead to tracheomalacia.

## MATERIALS AND METHODS

### Animal models

All mouse experiments were approved by the Institutional Animal Care and Use Committee at Cincinnati Children's Hospital Medical Center (CCHMC) under protocol 2019-0006. Animals were housed within the CCHMC Veterinary Services Core in temperature-controlled rooms with regular access to food and water. Mice were maintained on outbred background. Most embryos were harvested before overt sexual differentiation and analysis of the entire litter suggested an equal distribution of male and females. Dr Debora Sinner provided *Foxg1Cre* (Hébert and McConnell, 2000), *Dermo1Cre* (Sosic et al., 2003) and *mTmG* (Muzumdar et al., 2007) animals, as well as *Foxg1Cre;Sox9^f/f^* (Kist et al., 2002) mutant and control samples. Dr Vladimir Kalinichenko provided *Foxf1^fl/fl^* animals (Ren et al., 2014). Dr Samantha Brugmann (Cincinnati Children's Hospital, OH, USA) provided *Wnt1Cre* (Lewis et al., 2013), *mTmG* (Muzumdar et al., 2007) and *Gli3T^Flag/Flag^* (Vokes et al., 2008) animals. Dr Joo-Seop Park (Cincinnati Children's Hospital, OH, USA) provided *Gli3T^Flag/Flag^* animals (Vokes et al., 2008).

### Immunostaining, *in situ* hybridization and Alcian Blue staining

At least three embryos of each genotype were used for all experiments. For section immunostaining, embryos were collected and incubated in 4% paraformaldehyde solution overnight at 4°C. After two rinses in 1× PBS, embryos were incubated in 30% sucrose overnight at 4°C before embedding in optimal cutting temperature (OCT) compound for cryosectioning. Sections were collected at 8 µm. *Foxg1Cre;Sox9^f/f^* embryos were embedded in paraffin before sectioning, and were deparaffinized before immunostaining. On day 1 of immunostaining, sections were washed in 1× PBS before incubation in 1× PBS with 0.05% Triton X-100. Sections were then blocked with 5% normal donkey serum in 1× PBS for 1 h before overnight incubation in primary antibodies (Foxf1, goat, R&D Systems, AF4798, 1:300; anti-Sox9, mouse, Invitrogen, 14-9765-82, 1:200; anti-Sox9, rabbit, Millipore, AB5535, 1:200; Acta2, mouse, Sigma-Aldrich, A5228, 1:800; Acta2, rabbit, Genetex, GTX100034, 1:800; pHH3, mouse, Millipore, 05-1336, 1:1000; CC3, rabbit, Cell Signaling Technology, 9661, 1:200; GFP, chicken, Aviva Biosystems, GFP-1020, 1:1000; DsRed, mouse, Living Color, 632392, 1:1000; and anti-β-galactosidase/LacZ, chicken, Abcam, ab9361, 1:1000) at 4°C. On the second day, sections were washed three times in 1× PBS before incubation in secondary antibodies (donkey anti-mouse IgG Alexa Fluor 647, Jackson ImmunoResearch, 715-606-151; donkey anti-goat IgG Alexa Fluor 647, Jackson ImmunoResearch, 705-606-147; donkey anti-rabbit IgG Alexa Fluor 647, Jackson ImmuoResearch, 711-605-152; donkey anti-rat IgG Alexa Fluor 647, Jackson ImmunoResearch, 712-606-153; donkey anti-rabbit IgG Cy3, Jackson ImmunoResearch, 711-165-152; donkey anti-mouse IgG Cy3, Jackson ImmunoResearch, 715-165-151; donkey anti-goat IgG Alexa Fluor 488, Jackson ImmunoResearch, 705-546-147; donkey anti-chicken IgG Alexa Fluor 488, Jackson ImmunoResearch, 703-546-155; donkey anti-chicken IgG Alexa Fluor 647, Jackson ImmunoResearch, 703-606-155; donkey anti-rabbit IgG Alexa Fluor 405, Abcam, ab175649; and DAPI, Thermo Scientific; all at 1:500) at room temperature, and were washed in 1× PBS three more times before coverslip placement. All antibodies have been validated by numerous previous publications and, where practical, by loss of signal in genetic null mutants.

*In situ* hybridization was performed using an RNAScope Multiplex Fluorescent v2 kit according to the manufacturer's instructions (ACD Biosystems). For whole-mount immunostaining, embryos were stored in methanol at −20°C before beginning staining. Foreguts were dissected out, incubated in Dent's bleach for 2 h, and were serially rehydrated into 1× PBS before blocking in 5% normal donkey serum and 1% DMSO for 2 h. Foreguts were then incubated in primary antibody diluted in blocking solution overnight at 4°C. After five washes in 1× PBS, foreguts were incubated in secondary antibody overnight at 4°C. The next day, after three washes in 1× PBS, foreguts were serially dehydrated into methanol and stored at 4°C overnight before clearing in Murray's clear solution for imaging. All images were taken on a Nikon LUNA upright confocal microscope in the CCHMC Confocal Imaging Core.

Alcian Blue staining was performed on dissected foreguts as described previously (Que et al., 2007). Foreguts were then serially rehydrated into 1× PBS before incubation in 30% sucrose overnight at 4°C. After embedding in OCT, foreguts were cryosectioned at 60 µm and photographed using a Nikon LUN-A inverted widefield microscope.

### Quantitative analysis

Confocal images were analyzed using Nikon Elements Analysis and Imaris programs. All statistical analyses were performed in Microsoft Excel on data obtained from single transverse sections from each embryo. Sections were selected based on their median location between the anterior separation of the larynx into the trachea and the posterior formation of the mainstem bronchi from the trachea. Calculations were performed in Microsoft Excel using an unpaired two-sided Student's *t*-test with unequal variance and with significance defined as *P*<0.05. No specific power calculation was performed. The sample size is indicated in each figure and no data were excluded. The genotype of immunostaining results was blinded to co-investigators for interpretation. Graphs were generated using GraphPad Prism. For relative expression of Foxf1 and Sox9 in the tracheal mesoderm (referred to as TMes in figures), the number of Foxf1^+^ or Sox9+ tracheal mesoderm cells was divided by the total number of tracheal mesoderm cells. For pHH3^+^ (mitotic indices) or CC3^+^ rates of mesenchyme cells, the number of tracheal mesenchymal cells positive for either pHH3 or CC3 was divided by the total number of tracheal mesenchymal cells. For quantification of immunostaining, values are reported as mean±s.e.m., with *P*<0.05 as calculated by a two-sided Student's *t*-test with unequal variance.

### RNA-seq and ChIP-seq analysis

RNA-Seq analysis was performed on control and *Foxg1Cre;Gli3T^Flag/+^* samples sequenced at stages E10.5 (foreguts) and E11.5 (tracheas) with three independent biological replicates (embryo dissections) for each condition. After storing in RNALater (Ambion) at −80°C, RNA was isolated using a Qiagen MicroEasy Kit and was amplified by the CCHMC Gene Expression Core before sequencing in the CCHMC DNA Sequencing Core using an Illumina 3000 high-throughput platform. Single-end sequencing with read-depth of ∼22-27 million and read length of 75 bp was performed. Raw reads from the experiments were analyzed using Computational Suite for Bioinformaticians and Biologists (CSBB -v3.0, https://github.com/praneet1988/Computational-Suite-For-Bioinformaticians-and-Biologists). The following steps were carried out in analysis using CSBB. Quality check and trimming was performed using FASTQC (www.bioinformatics.babraham.ac.uk/projects/fastqc/) and Bbduck (www.jgi.doe.gov/data-and-tools/bbtools/bb-tools-user-guide/bbduk-guide/), respectively. Quality trimmed reads were then mapped to the mouse genome (mm10) using Bowtie2, and quantified using RSEM (https://bmcbioinformatics.biomedcentral.com/articles/10.1186/1471-2105-12-323).

Differential expression analysis was carried out using CSBB-Shiny (https://github.com/praneet1988/CSBB-Shiny), and volcano plots were generated using the EnhancedVolcanco package (www.bioconductor.org/packages/release/bioc/vignettes/EnhancedVolcano/inst/doc/EnhancedVolcano.html) in R. Differentially expressed genes were obtained at the following thresholds: LogFC≥|1| and 0.05≥False Discovery Rate.

ChIP-Seq analysis was performed on the following published datasets: (1) Foxf1 ChIP on dissected E18.5 lung (GSE77159, Dharmadhikari et al., 2016); (2) Gli3-3xFlag ChIP on dissected E10.5 limb buds (GSE133710, Lex et al., 2020); and (3) ATAC-seq and H3K4me3 ChIP performed on E9.5 cardiopulmonary progenitors (GSE119885, Steimle et al., 2018). These datasets were reprocessed using CSBB, and for visualization purposes, bigwig files were generated using deepTools (BamCoverage function) (Ramírez et al., 2016) from bam files. Peaks were called using Macs2 (default parameters) (Zhang et al., 2008). Genome browser views were generated using IGV.

## Supplementary Material

Supplementary information
