# Supplementary material for: Disruption of a Hedgehog-Foxf1-Rspo2 signaling axis leads to tracheomalacia and a loss of Sox9+ tracheal chondrocytes
Source: Dis Model Mech. 2021 Feb 4;14(2):dmm046573. doi: 10.1242/dmm.046573 (PMC7875488; doi:10.1242/dmm.046573)
Supplement: Supplementary information [file dmm-14-046573-s1.pdf]

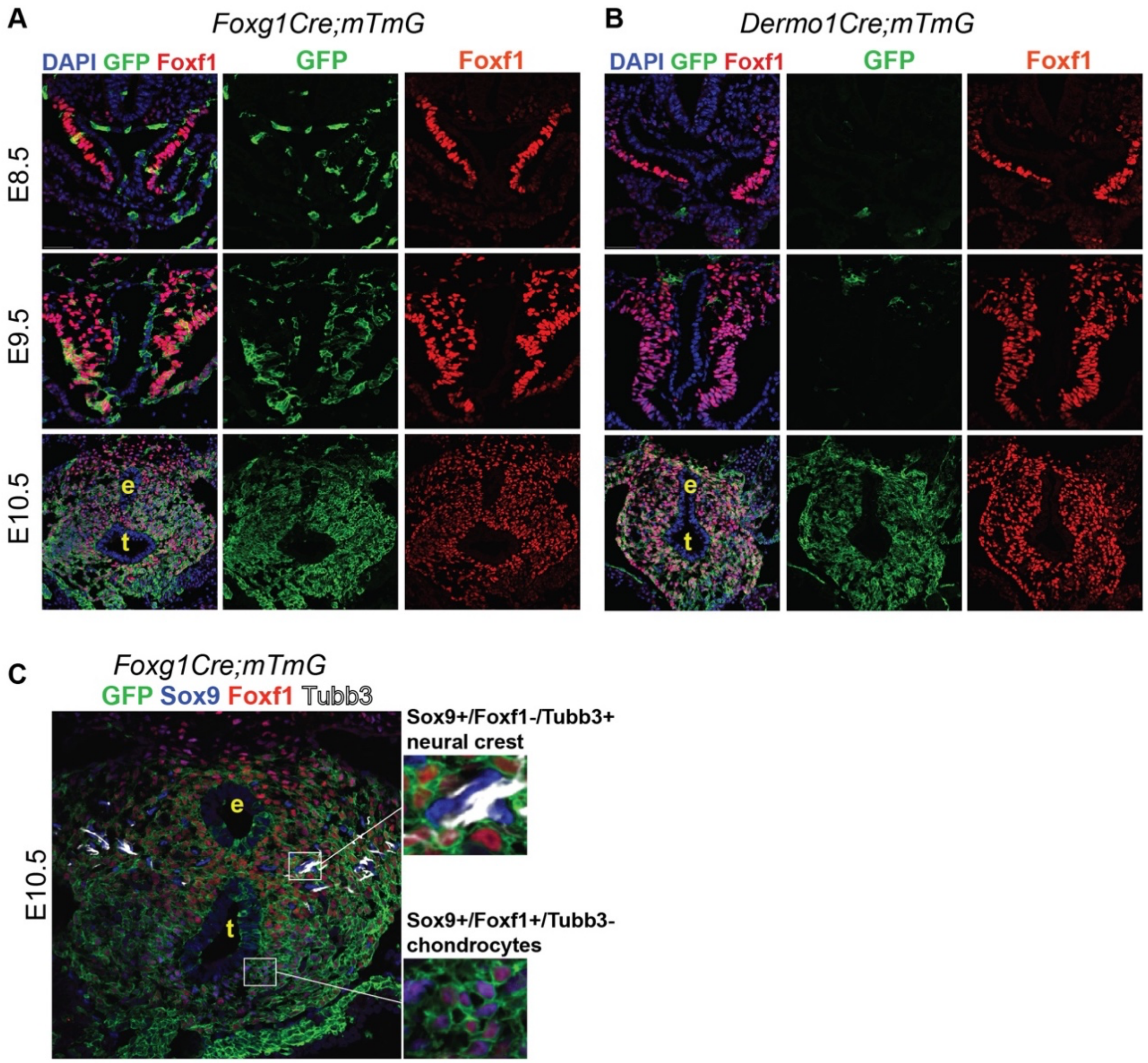

Figure S1: Kinetics of Foxg1Cre and Dermo1Cre Recombination
(A-B) GFP and Foxf1 immunostaining of (A) *Foxg1Cre;mTmG* and (B) *Dermo1Cre;mTmG* foreguts and E8.5, E9.5,and E10.5. The Foxg1Cre line starts to recombine at E8.5 with robust recombination at E9.5, whereas the Dermo1Cre starts to recombine at E9.5 with robust recombination by E10.5. (C) Immunostaining of E10.5 *Foxg1Cre;mTmG* embryos showing GFP-/Sox9+/Foxf1-/Tubb3+ neural crest cells in surrounding the esophagus are distinct from the GFP+/Sox9+/Foxf1+Tubb3- chondrocytes surrounding the ventral trachea. All scales bars 100 µm. N=2-3 embryos/ genotype/stage.

Disease Models & Mechanisms • Supplementary information

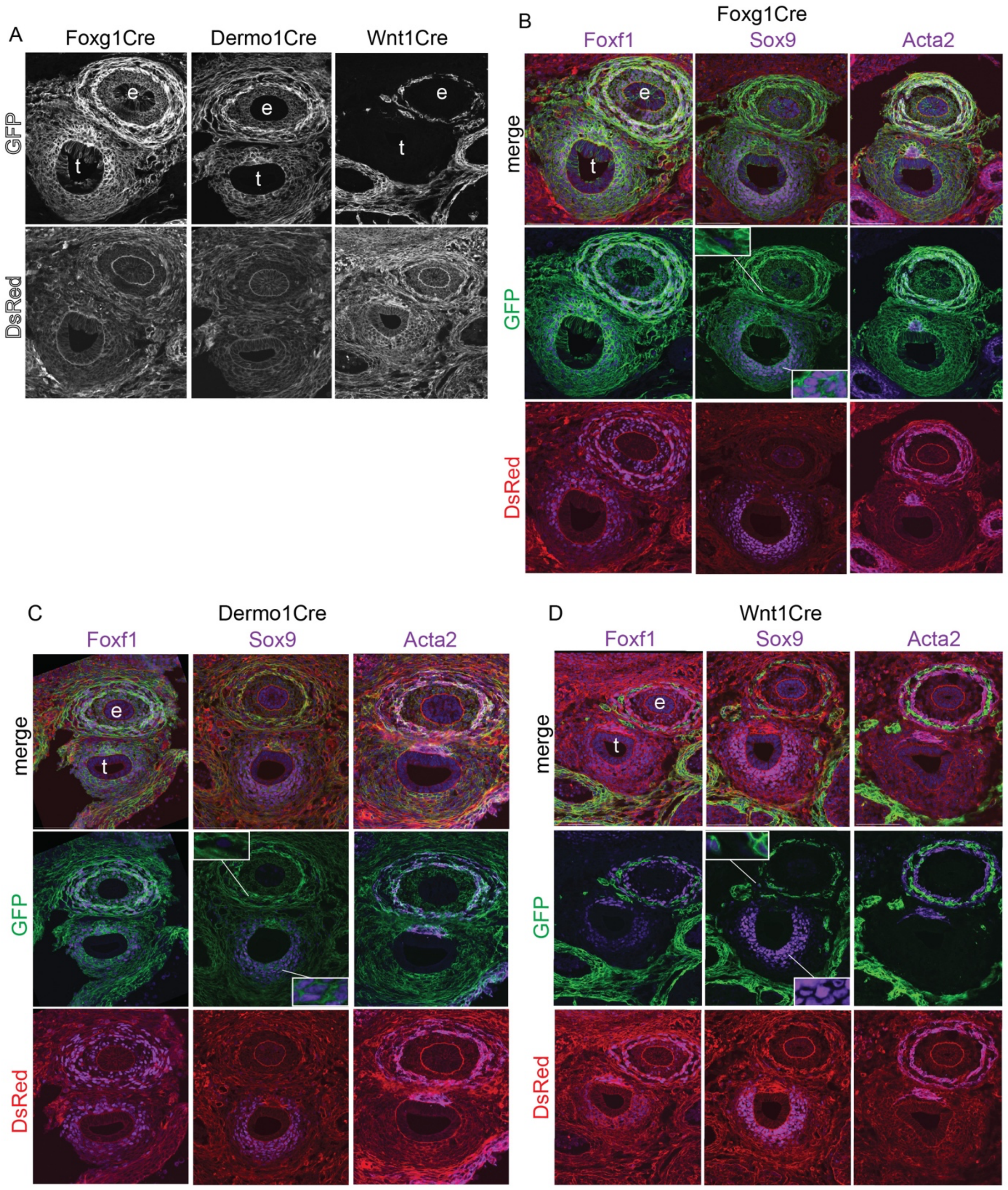

Figure S2: *Foxg1Cre*, *Dermo1Cre*, and *Wnt1Cre* Lineage Tracing in Tracheoesophageal Development

(A) GFP and DsRed immunostaining of E13.5 *Foxg1Cre;mTmG*, *Dermo1Cre;mTmG*, and *Wnt1Cre;mTmG* foreguts show that as expected the *Foxg1Cre* and *Dermo1Cre* lineages give rise to tracheoesophageal mesenchyme, while the Wnt1 neural crest cell lineage gives rise to neurons that innervate the esophageal and trachealis muscles. (B-D) Immunostaining of GFP and DsRed with Foxf1, Sox9, or Acta2 in (B) *Foxg1Cre;mTmG* embryos, (C) *Dermo1Cre;mTmG* embryos and (D) *Wnt1Cre;mTmG* embryos at E13.5. Insets show that the *Foxg1Cre* and *Dermo1Cre* linage trace the lateral plate mesoderm that gives rise to GFP+/Foxf1+/Acta2+ smooth muscle and GFP+/Sox9+ chondrocytes, but not the GFP-/Sox9+ enteric neurons between the esophageal smooth muscle. In contrast neural crest specific *Wnt1Cre* lineage traces the Sox9+ cells that are likely to be enteric neurons innervating the esophageal and tracheal smooth muscle. All scale bars, 100 μm. e=esophagus, t=trachea. N= 3 embryos / genotype

Disease Models & Mechanisms • Supplementary information

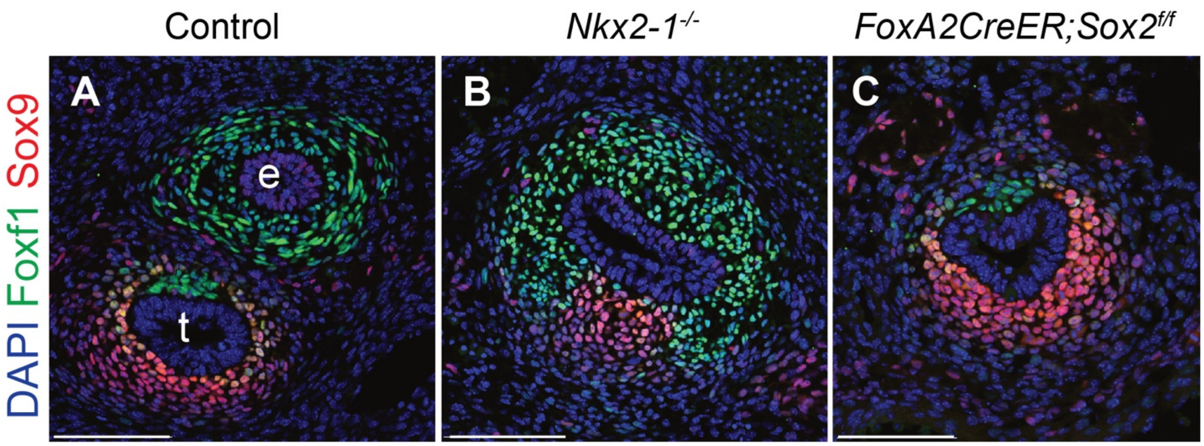

Figure S3: Foxf1 and Sox9 Expression in *Sox2* and *Nkx2-1* Mutants
(A-C) Immunostaining of Foxf1 and Sox9 at E13.5 in (A) control, (B) *Nkx2-1*$^{GFP/GFP}$ (or *Nkx2-1*$^{-/-}$) and (C) *FoxA2CreER;Sox2*$^{f/f}$. *Nkx2-1* and *Sox2* mutants both have a single unseparated foregut tube that exhibits appropriate dorsal-ventral patterning. However *Nkx2-1* mutants have fewer Sox9+ chondrocytes whereas *Sox2* mutants have fewer Foxf1+ smooth muscle progenitors. Scale bar = 100 μm. e = esophagus, t = trachea. N = 3 embryos / genotype.

Disease Models & Mechanisms • Supplementary information

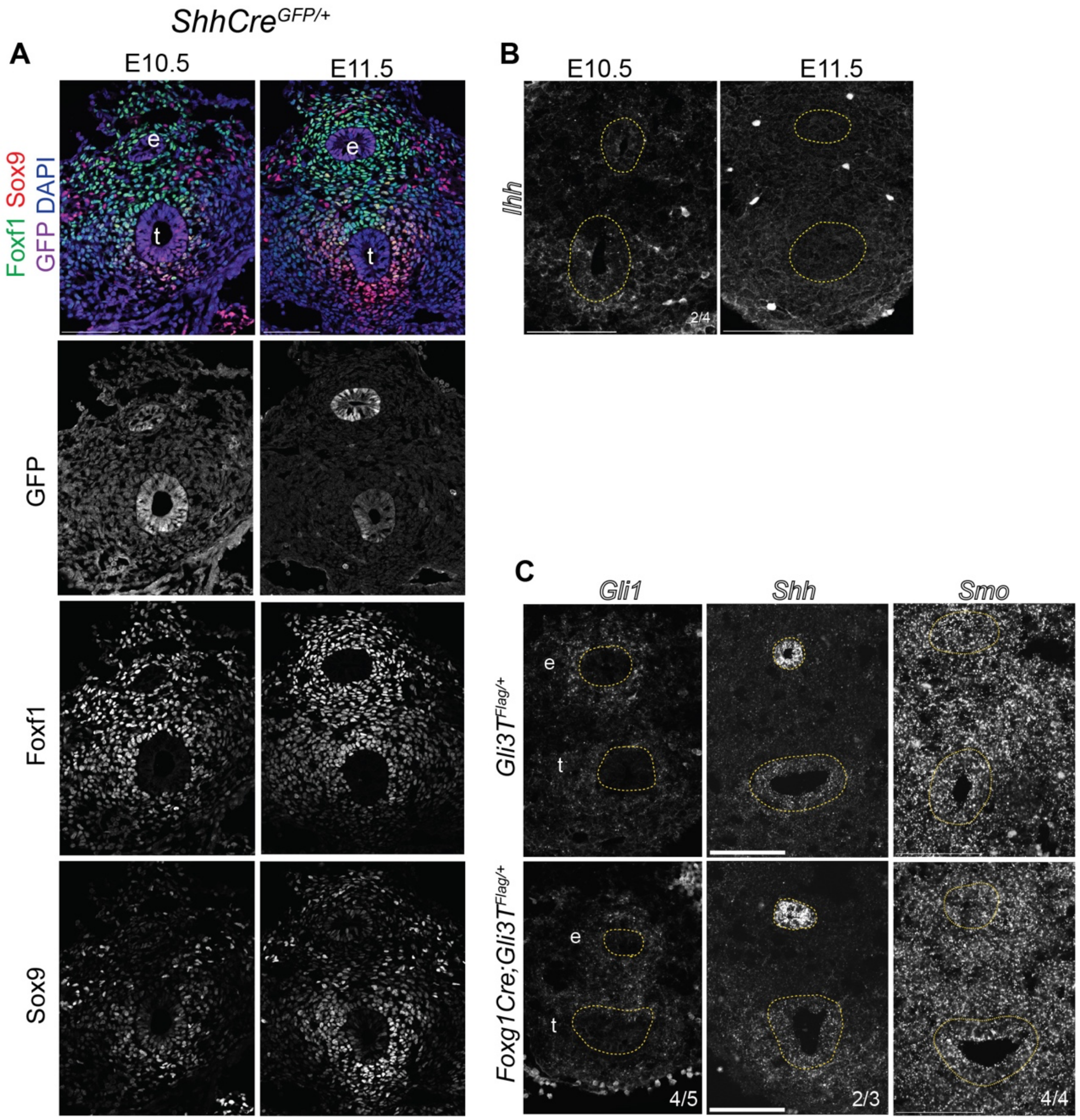

Figure S4: Expression of HH Pathway Components

(A) Immunostaining of *ShhCre*$^{GFP/+}$ heterozygous embryos (Cre-GFP fusion knocked into the endogenous *Shh* loci). GFP immunostaining shows a shift in *Shh* expression from trachea at E10.5 to esophageal at E11.5. (B) RNAscope *in situ* hybridization of Ihh at E10.5 and E11.5. (C) RNAscope *in situ* hybridization of *Gli1*, *Shh*, and *Smo* in E11.5 control and *Foxg1Cre;Gli3T*$^{Flag/+}$ embryos. Scale bar = 100 μm. e = esophagus, t = trachea. N = 3-5 embryos / genotype.

Disease Models & Mechanisms • Supplementary information

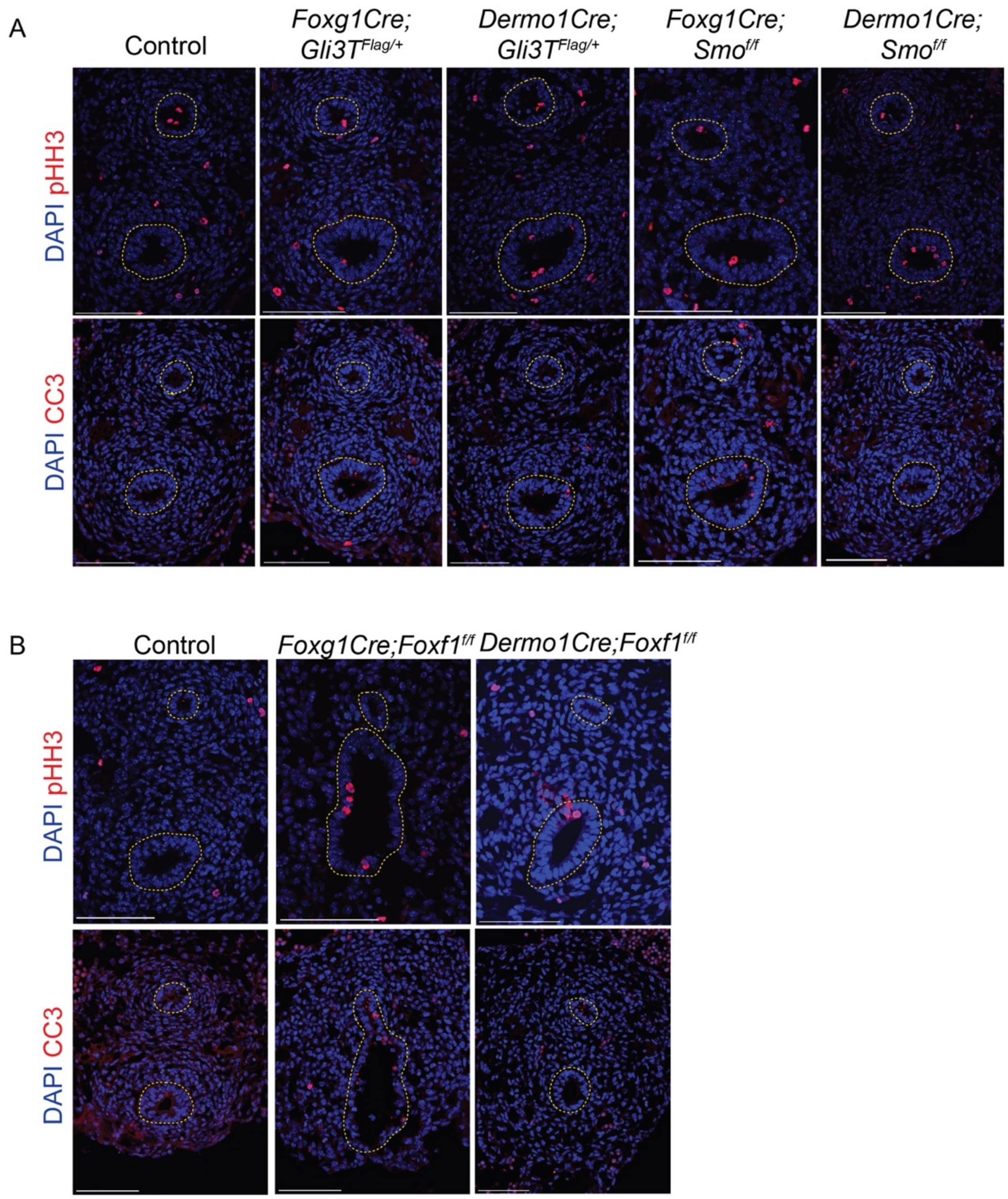

Figure S5: Cell Proliferation and Apoptosis in HH/Gli and Foxf1 Mutants

(A-B) Immunostaining of cell proliferation marker phospho-Histone H3 (pHH3) or cell apoptosis marker cleaved caspase-3 (CC3) in (A) E11.5 control and *Gli3T*$^{Flag/+}$ and *Smo* mutants or (B) in E11.5 control and *Foxf1* mutants There is no substantial difference in proliferation or cell death in the any mutants compared to control embryos. N=3-5 embryos/genotype.

Disease Models & Mechanisms • Supplementary information

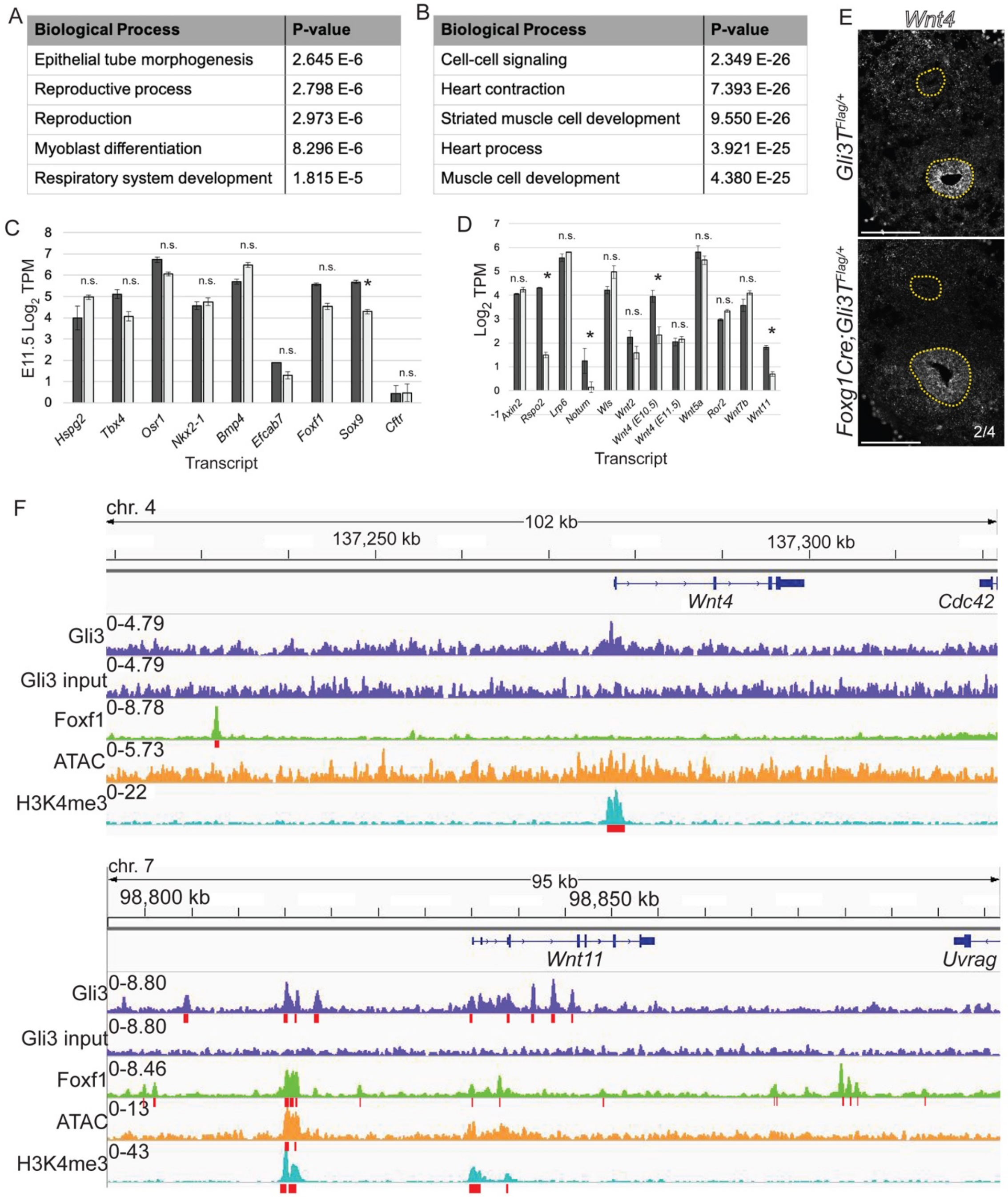

A

Biological Process	P-value
Epithelial tube morphogenesis	2.645 E-6
Reproductive process	2.798 E-6
Reproduction	2.973 E-6
Myoblast differentiation	8.296 E-6
Respiratory system development	1.815 E-5

B

Biological Process	P-value
Cell-cell signaling	2.349 E-26
Heart contraction	7.393 E-26
Striated muscle cell development	9.550 E-26
Heart process	3.921 E-25
Muscle cell development	4.380 E-25

Figure S6: Additional Data on Gli3 Target Genes.
(A-B) GO enrichment analysis of (A) downregulated and (B) upregulated genes in *Foxg1Cre;Gli3T$^{Flag/+}$* mutants (E10.5 and E11.5 combined).(C-D) Histogram of RNA-seq transcript levels in E11.5 trachea tissue showing (C) selected mediators of tracheal chondrogenesis and (D) Wnt-pathway genes. Control expression in black and mutant expression in grey. Asterisks indicate differential expression of $Log_2TPM \geq |1|$, $p<0.05$ in E11.5 *Foxg1Gli3T$^{Flag/+}$* tracheas. (E) Genome browser views of Gli3-3xFlag (GSE133710), Foxf1 (GSE77159), and H3K4me3 (GSE119885) ChiP-seq data, as well as ATAC-seq data (GSE119885) in the *Wnt4* and *Wnt11* loci. *Wnt4* appears to be a direct Foxf1 target, while *Wnt11* may be directly regulated by both Gli3 and Foxf1. (F) RNAscope *in situ* hybridization of *Wnt4* in E11.5 control and *Foxg1Cre;Gli3T$^{Flag/+}$* tracheas.

Disease Models & Mechanisms • Supplementary information

Table S1

Click here to Download Table S1

Table S2

Click here to Download Table S2
